# Hysteria

**Published:** 1860-05

**Authors:** J. P. Ross

**Affiliations:** Attending Physician to City Hospital


### HYSTERIA.

REPORTED BY J. P. ROSS, M. D., ATTENDING PHYSICIAN TO CITY HOSPITAL.

Emeline E., aged twelve years, was admitted into the City Hospital January 20th, 1860. At the time of her admission it was impossible to obtain a correct history of the case from the patient herself, but the following statement, given by her mother, a very intelligent woman, may be relied on as correct. She states that no hereditary tendency to disease exists in the family; that Emeline has always enjoyed good health until December, 1858, at which time she dates the commencement of her present illness. At that time she was descending a stairway and fell the distance of a few feet, striking on the lower part of the spinal column. The injury was so slight as not to attract attention at the time, and nothing was thought of it until the spring of 1859, when the family physician was consulted. At this time she complained of a pain in the back and loins, habitual constipation, frequent headache, and considerable impairment of the motion of the lower extremities. The attending physician made a careful examination of the case, and, without promising any relief, ordered counter-irritation to the spine, with internal medicines, and occasional purges to move the bowels. This treatment was sedulously employed for six weeks without any apparent relief. During this time the bowels became so obstinately constipated that it required the most active medicines to produce the desired effect, and each movement was attended with severe pain and tenesmus. There was also occasional retention of urine, and convulsions, with manifest impairment of the mental faculties. This condition of things induced the attending physician to solicit an examination per rectum and per vaginum, which, however, it was impossible to obtain without using force with the patient.

The examination revealed a mal-position of the womb in such a way that pressure was maintained on the rectum and bladder, preventing those organs from discharging their functions. The treatment was now somewhat changed. Counter-irritation to the spine was discontinued and such means used as were calculated to correct the displacement of the womb. This plan of treatment was continued three weeks, during which time all the symptoms were aggravated, and in addition, there was complete loss of motion of the lower extremities, the legs being flexed upon the thighs, and the thighs upon the abdomen, and constantly retained in that position. The displacement of the womb had been frequently reduced by the attending physician, and such remedies used as were calculated to retain the organ in its proper position—such as astringent injections and tonics. At no time during these manipulations was there any appearance of an os utero, and as a consequence a malformation of the organ was suspected. This suspicion, together with the complete inefficiency of the means thus far employed, induced the attending physician to ask council, which being readily acceeded to by the friends of the patient, a consultation was held June 10th, 1859. The treatment after the consultation was somewhat modified, the consulting physician being unable to make out any serious displacement or malformation of any kind. Rest, soothing injections, and tonics were the principal means recommended and used for several weeks, but without any beneficial result. It was then thought she was the subject of nymphomania which kept up in some way the exciting cause of all this difficulty. A strict watch was instituted to detect her, if possible, in the act of violating her person, but failing in this, and finding the symptoms becoming aggravated it was deemed advisable to employ additional council. A second consultation was called and a careful examination instituted. At this time it was thought the difficulty was principally an enlarged and calloused condition of the os uteri. The solid stick of the nitrate of silver was applied to the diseased organ, and its use recommended every second day. The application of the caustic was attended with excruciating pain, which lasted for twelve hours, and as no good resulted therefrom, its further use was dispensed with. The same plan of treatment as before the last consultation was continued with all diligence and care for the next three months, during which time the condition of the patient became truly miserable, and without any hope of relief from any source, she was sent to the City Hospital, having been under treatment nearly nine months.

She came under my care February 1st, 1860. At this time the general nutrition of the body was good, with, perhaps, the exception of the lower extremeties. These were closely flexed upon the body, and entirely useless. No kind of persuasion could induce her to make any effort to extend them. The muscles were flaccid and somewhat emaciated, but in other respects normal. Her disposition was exceedingly erratic. At times she would cry like a child, and then becoming suddenly angry, would curse, with horrid oaths, her attendants. Her physical strength was unimpaired. A careful examination of the spine revealed nothing abnormal; there was no tenderness or deformity in any part of its length. The thoracic and abdominal organs were all healthy. An examination of the uteris was not deemed necessary. The length of time the disease had existed—the entire absence of any organic lesion—the well nourished condition of the body—together with the great perversion of the mental faculties—forced me to the conclusion that I had to deal with a case of simple hysteria, disconnected with any organic disease, whatever.

Regarding the case then as one of simple hysteria, I did not adopt any plan of active treatment. The cautics, the injections, the active purges, and the tonics, were discontinued. No medicine was given. The Matron of the Hospital, a very capable woman, was asked to take charge of the case and give the advice such persons require, and if possible get her rid of the idea that she was a dying invalid. She kindly consented to do this for me, and the result has been most satisfactory.

February 15th.—Patient up and walking about for the first time; makes no complaints, and requires no attention at my hands; is still a little irritable.

March 1st.—Discharged cured.

*Remarks.*—However accurately this case simulated all the diseases—and many more—for which she was treated before she entered the Hospital, it is very evident that her whole trouble had a mental origin. This is made clear by, not only the previous history, but also the result of the treatment after her admission into the Hospital. When she entered the Hospital she was in no wise better than she had been at any time for the previous nine months. The loss of motion in the lower extremities was complete, and the mental derangement so entire, that she seemed on the border of hopeless insanity. Now how much the idea which had become fixed in her mind, that she could not control the paralysis and mental derangement, had to do with the difficulty, is made evident by dispelling this idea and fixing the opposite; for, as fast as confidence came that she could walk, and control her mental aberrations, she possessed and exercised the power to do both; and as mental harmony was restored, physical health and harmony followed as a sequence. I suppose the uterus had very little to do with the case as a *cause* of the difficulty. If the efforts of nature to establish the menstrual function was at the bottom of the trouble—which is a more natural view than many that have been entertained—the health would not have been restored until this function was established. Yet she was discharged in perfect health, without the slightest show of the menses appearing at any time.

